# Comparison of two different uses of underbody forced-air warming blankets for the prevention of hypothermia in patients undergoing arthroscopic shoulder surgery: a prospective randomized study

**DOI:** 10.1186/s12871-022-01597-6

**Published:** 2022-02-28

**Authors:** Wenchao Yin, Qihai Wan, Haibin Jia, Xue Jiang, Chunqiong Luo, Lan Zhang

**Affiliations:** Department of Anesthesiology, Sichuan Provincial Orthopedic Hospital, No. 132 West First Section First Ring Road, Chengdu, 610041 Sichuan China

**Keywords:** Forced-air warming, Core body temperature, Inadvertent perioperative hypothermia, Arthroscopic shoulder surgery

## Abstract

**Background:**

Forced-air warming (FAW) is an effective method of preventing inadvertent perioperative hypothermia (IPH). However, its warming effects can be influenced by the style and position of the FAW blanket. This study aimed to compare the effects of underbody FAW blankets being placed under or over patients in preventing IPH.

**Methods:**

Patients (*n*=100) undergoing elective arthroscopic shoulder surgery in the lateral decubitus position were randomized into either under body (UB) group or the over body (OB) group (50 per group). The body temperature of the patients was recorded from baseline to the end of anesthesia. The incidences of postoperative hypothermia and shivering were also collected.

**Results:**

A steady decline in the body temperature was observed in both groups up to 60 minutes after the start of FAW. After 60 minutes of warming, the OB group showed a gradual increase in the body temperature. However, the body temperature still decreased in UB group until 75 minutes, with a low of 35.7℃ ± 0.4℃. Then the body temperature increased mildly and reached 35.8℃ ± 0.4℃ at 90 minutes. After 45 minutes of warming, the body temperature between the groups was significantly different (*P* < 0.05). The incidence of postoperative hypothermia in the UB group was significantly higher than that in the OB group (*P* = 0.023).

**Conclusions:**

The body temperature was significantly better with the use of underbody FAW blankets placed over patients than with them placed under patients. However, there was not a clinically significant difference in body temperature. The incidence of postoperative hypothermia was much lower in the OB group. Therefore, placing underbody FAW blankets over patients is recommended for the prevention of IPH in patients undergoing arthroscopic shoulder surgery.

**Trial registration:**

This single-center, prospective, RCT has completed the registration of the Chinese Clinical Trial Center at 13/1/2021 with the registration number ChiCTR2100042071. It was conducted from 14/1/2021 to 30/10/2021 as a single, blinded trial in Sichuan Provincial Orthopedic Hospital.

**Supplementary Information:**

The online version contains supplementary material available at 10.1186/s12871-022-01597-6.

## Introduction

Inadvertent perioperative hypothermia (IPH), defined as core body temperature (CBT) <36℃, is still common in surgery, with a reported incidence of more than 40% [1, 2]. IPH can be induced by numerous factors, such as thermoregulatory impairment, low ambient temperature in the operating room, room temperature infusion and irrigation fluids, and surgical exposure [1, 3, 4]. Among these factors, thermoregulatory impairment induced by anesthesia is by far the most important [3]. In addition to increasing discomfort, IPH has been proposed to be associated with a series of adverse events in patients [5], such as wound infections [6, 7], myocardial events [3, 5], reversible coagulopathy [3], postoperative delirium [8], poor wound healing [9], and increased blood loss [3, 10]. These adverse events lead to prolonged hospitalization, and some may even affect a patient’s long-term quality of life.

In clinical practice, IPH has been an ongoing challenge for perioperative team members. Many medical specialty societies, such as the National Institute for Health and Care Excellence (NICE) (www.nice.org.uk) and the Association of Perioperative Registered Nurses (AORN) (www.aorn.org) have developed guidelines for IPH prevention. These guidelines recommend measuring and monitoring the patient’s temperature throughout the perioperative period [11, 12]. They also give advice on how to select and implement warming interventions to prevent or treat IPH [11, 12]. Providing timely and proper warming interventions for patients can effectively prevent or treat IPH, and further reduce the incidence of complications induced by IPH.

A number of patient-warming devices have been developed to prevent IPH and may be broadly classified into conductive heating or convective heating systems based on their mechanism of heat generation. The most commonly used conductive heating system is a resistive heating system, which typically involves placing a reusable resistive heating mattress under patients to generate a uniform heating surface [7, 13]. As the only convective heating system used during surgery, a forced-air warming (FAW) system delivers warmed air through a disposable blanket. Studies have demonstrated that the FAW system is superior to resistive heating systems and any other active warming methods for the prevention of IPH in patients undergoing surgery [7, 14]. Additionally, the use of a resistive heating mattress may affect ECG monitoring [15]. Therefore, although the use of FAW has ongoing and cumulative costs, FAW is still widely used to prevent IPH during surgery.

Previous studies have shown the warming effects of FAW may be affected by the FAW blanket style and position [16–18], and suggested that the largest blanket that is possible for the operation should be used [18]. During arthroscopic shoulder surgery, as during any other surgeries that require special surgical positioning of patients, anesthesiologists and operating room nurses prefer to place a full access FAW blanket under the patient's body rather than over it because of the ease of setup and management. However, studies on the efficacy of the two aforementioned FAW blanket placements are scarce despite patients being susceptible to IPH during arthroscopic shoulder surgery. Therefore, this study was designed to determine how to most effectively apply FAW in patients undergoing arthroscopic shoulder surgery.

## Methods

### Ethics

This study was conducted in accordance with the Declaration of Helsinki tenets, and approved by the Ethics Committee of Sichuan Provincial Orthopedic Hospital on August 22, 2020 (reference KY2020-001-01). The study was registered at www.chictr.org.cn on January 13, 2021 with the registration number ChiCTR2100042071.

### Study population

The study adhered to the Consolidated Standards of Reporting Trials (CONSORT) guidelines [19]. Eligible participants were identified from among the patients scheduled for elective arthroscopic shoulder surgery. We included patients with American Society of Anesthesiologists (ASA) Physical Status I or II, an age of 18 to 70 years, and an expected duration of surgery longer than 80 min. We excluded patients who declined to participate in the study and those with thyroid dysfunction or severe vascular disease, body mass index (BMI) >30 kg/m^2^ and contraindications to peripheral nerve block. Patients were enrolled after signing written informed consent forms. A computer-generated, 1:1 ratio block randomization schedule was used, and the sequentially numbered, sealed, opaque envelopes containing the assignments were prepared by a nurse who was not otherwise involved in the study. Enrolled patients were assigned to either the under body (UB) or over body (OB) group according to the number in their envelope, which was opened by the anesthesiologist immediately after the patient arrived in the preoperative waiting area.

### Procedures

In this study, anesthesia was performed by a single anesthesiologist, and all surgeries were performed by the same surgical team**.** The ambient temperature of the operating room was maintained at 21℃ ± 1℃. Room temperature normal saline solution in a 3-L bag was used as irrigation fluid. The gravity flow system consisted of 2 saline bags suspended 70 cm above the operative region to create an inflow pressure for adequate intra-articular visualization. Sometimes, to improve the visualization, we permitted the inflow pressure to be elevated by raising the height of the saline bags for brief periods.

On arrival in the operating room, the baseline temperature of the patient (T_a_) was recorded. Intravenous midazolam (1 to 2 mg) was administered for sedation under standard ASA monitoring and supplemental oxygen. Then the patients received an interscalene nerve block by using a combination of ultrasonographic visualization and neurostimulation with 20 mL of 0.2% ropivacaine. After the completion of block, the patient’s temperature (T_b_) was collected, and then all patients underwent general anesthesia with tracheal intubation using IV propofol, rocuronium, and IV sufentanil with conventional clinical doses. Anesthesia was maintained with continuous infusion of remifentanil and inhalation of sevoflurane. Patients were subsequently placed in the lateral decubitus position with the operative-side shoulder above.

**Fig. 1:**
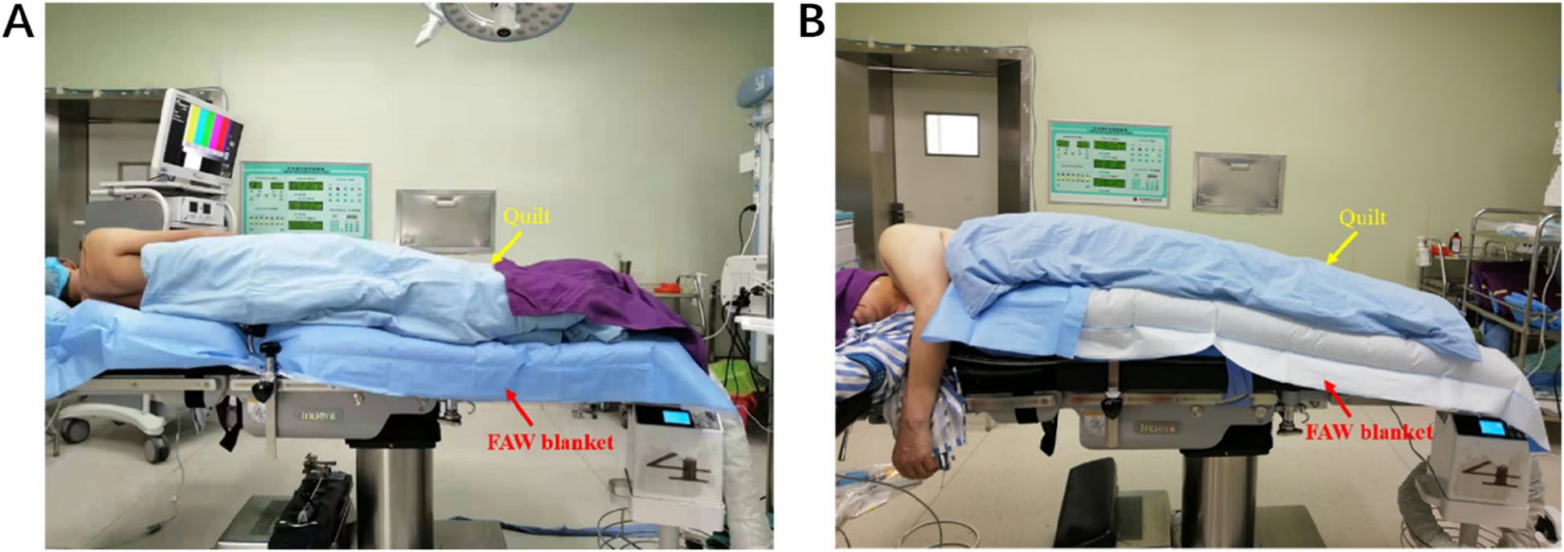
Patients were warmed using the forced-air warming (FAW) systems. **A**, FAW blanket was placed under the body of patient (UB group). **B**, FAW blanket was placed over the body of patient (OB group)

Patients in the UB group were warmed using a full access underbody FAW blanket (IOB-006, Jiangmen Dacheng Medical, China) that was placed under the patients in advance and in conjunction with an IOB heater (WU-505, Jiangmen Dacheng Medical, China) set at 43℃. According to the manufacturer’s guidelines, the full access underbody FAW blanket could also be used to cover the patients with the heating surface placed toward the patients during clinical use, if necessary. Therefore, to minimize confounding variables, patients in the OB group were also warmed with the above FAW units, but the FAW blanket was placed over the patients with the exception of the surgical area. The perforated side (the blue side with small holes) of the FAW blanket was placed towards the patients in both groups. In this study, the IOB heater was kept at the patients'feet, and all patients were covered with a common hospital quilt according to standard practice (Fig. 1). After disinfection and sterile towel coverage of the surgical area, the temperature of the patient (T_0_) was recorded, and the FAW unit was initiated. Subsequently, the temperature collection was performed at 15- minute intervals (T_15_, T_30_, T_45_…) until the end of the operation. After the patients had recovered from the anesthesia, we documented the postoperative body temperature and the incidence of hypothermia (< 36℃) and shivering. During the study, if the patient’s temperature was lower than 35℃ or higher than 38℃, we would terminate the study and take appropriate measures, such as stopping warming or providing warming in combination with other warming methods to maintain normal body temperature.

### Body temperature measurement

The iThermonitor WT705 (Raiing Medical, Beijing, China) is a non-invasive, wearable wireless thermometer that continuously measures the axillary temperature, and accurately estimates CBT [20, 21]. We applied it to monitor body temperature according to manufacturer’s guidelines and the method described in previous studies [20, 21]. In brief, the iThermonitor sensor was attached to the axilla on the non-operative side using a hypoallergenic adhesive patch. The sensor wirelessly transmitted the body temperature estimates to a hand-held terminal. Actually, the temperature reading on the hand-held terminal is the axillary estimates of CBT, and it can well represent the CBTs of patients [20, 21]. In addition, to ensure the accuracy of the temperature measurement, intravenous infusion was only allowed through the leg.

### Statistical analysis

The G*Power program (version 3.1) was used to calculate the sample size. A clinically significant difference in the temperature (primary outcome variable) between study groups was set at 0.6 °C according to Ralte et al.’s study with a standard deviation of 0.6 °C [7]. A total of 100 patients (50 per group), including 10% dropouts, was anticipated to provide 80% power at a significance level (α) of 0.05.

SPSS statistics 21.0 (IBM Corp, Armonk, NY, USA) was used for all statistical analyses in this study, and *P* values less than 0.05 were considered as statistically significant. Normally distributed continuous data are expressed as the mean ± SD, and the independent-samples t test was used to compare variables between study groups. We applied χ2 or Fisher exact tests to investigate associations among discrete variables.

## Results

**Fig. 2:**
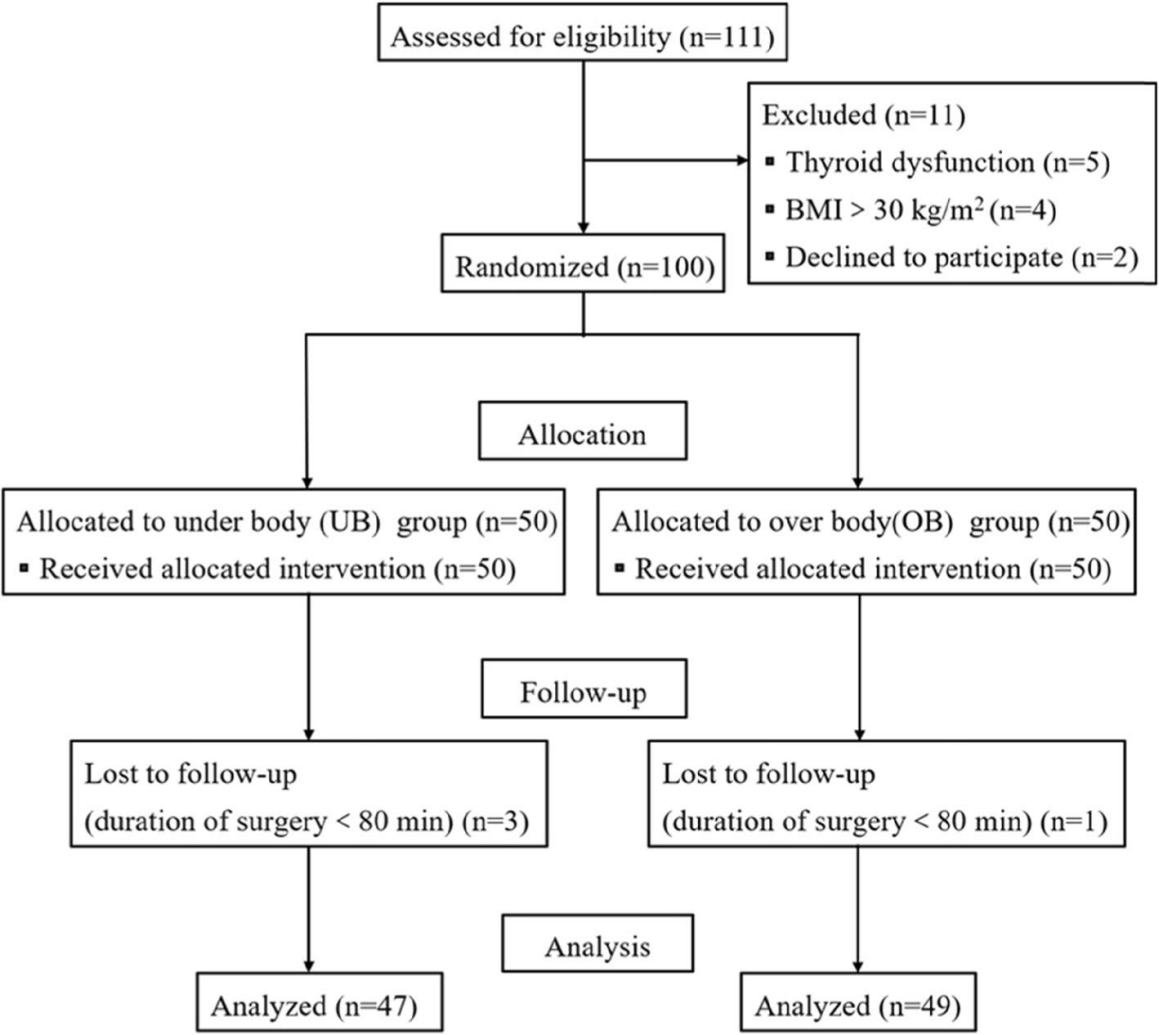
Consolidated Standards of Reporting Trials (CONSORT) diagram of patient flow through the study. BMI, body mass index

From January 14, 2021 to October 30, 2021, 111 patients who were scheduled for arthroscopic shoulder surgery were considered for eligibility, and 11 patients were excluded before randomization. A total of 100 patients were randomly allocated to one of the two groups (50 per group). We excluded three patients from the UB group and one patient from the OB group because the duration of surgery was shorter than 80 minutes which led to insufficient collection of temperature data. Therefore, data from 96 patients (47 in the UB group and 49 in the OB group) were analysed (Fig. 2). In some cases, although the intraoperative body temperature record exceeded 90 minutes, we only analysed the data collected up to 90 minutes.

**Fig. 3:**
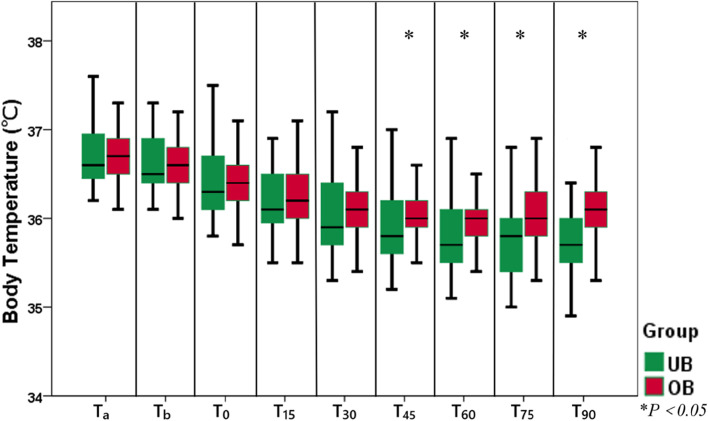
Body temperature variation during the procedure of surgery in underbody (UB) group and overbody (OB) group. Timepoints: T_a_ = baseline, T_b_ = before general anesthesia, T_0_ = pre-warming, T_15_ = after 15 min warming, T_30_ = after 30 min warming, T_45_ = after 45 min warming, T_60_ = after 60 min warming, T_75_ = after 75 min warming, T_90_ = after 90 min warming

Patient demographic characteristics (sex, age, BMI), infusion volume, irrigation time and volume are summarized in Table 1. No significant difference was noted between the two groups. The temperature variation during the procedure for both groups is shown in Table 2 and Fig. 3. There were no statistically significant differences in T_a_, T_b_ or T_0_ between the groups. In both groups, the body temperature declined rapidly after the induction of general anesthesia, and maintained a steady decline until 60 minutes after the start of FAW. From 60 minutes onward, a gradual increase was noted in the temperatures of patients in the OB group, reaching 36.1℃ ± 0.4℃ at 90 minutes. In the UB group, the body temperature exhibited a continuous and gradual decline until 75 minutes after the start of FAW, reaching a low of 35.7℃ ± 0.4℃. Then the body temperature increased mildly, and reached 35.8℃ ± 0.4℃ at 90 minutes. After 45 minutes of warming, compared with the UB group, the OB group had a significantly higher mean body temperature (*P*<0.05).

**Table 1:** Patient demographic characteristics, infusion volume, irrigation time and volume

	**UB group (*n*=47)**	**OB group (*n*=49)**	***P* value**
**Sex, male/female**	25/22	22/27	0.416
**Age, y**	51.7±12.9	52.1±12.4	0.877
**BMI, kg/m**^**2**^	24.8±2.9	24.1±2.9	0.231
**Infusion volume, mL**	761.7±148.3	752.0±141.4	0.745
**Irrigation time, min**	90.7±16.5	91.7±14.8	0.880
**Irrigation volume, L**	19.7±5.5	18.4±5.1	0.522

Data are expressed as Mean ± SD

**Table 2:** Comparison of body temperature during the procedure of surgery

Timepoints	Temperature (℃)		*P* value*
	UB group (*n*=47)	OB group (*n*=49)	
T_a_	36.7 ± 0.4	36.7 ± 0.3	0.881
T_b_	36.6 ± 0.4	36.7 ± 0.3	0.549
T_0_	36.4 ± 0.4	36.4 ± 0.4	0.907
T_15_	36.2 ± 0.4	36.3 ± 0.4	0.286
T_30_	36.0 ± 0.4	36.2 ± 0.4	0.101
T_45_	35.9 ± 0.4	36.1 ± 0.4	0.01
T_60_	35.8 ± 0.4	36.0 ± 0.4	0.003
T_75_	35.7 ± 0.4	36.1 ± 0.4	<0.001
T_90_	35.8 ± 0.4	36.1 ± 0.4	<0.001

Data are expressed as Mean ± SD. Timepoints: T_a_ = baseline, T_b_ = before general anesthesia, T_0_ = pre-warming, T_15_ = after 15 min warming, T_30_ = after 30 min warming, T_45_ = after 45 min warming, T_60_ = after 60 min warming, T_75_ = after 75 min warming, T_90_ = after 90 min warming

^*^The *P* value for the *t* test is set at 0.05

The postoperative temperature in patients in the OB group was significantly higher than that in the UB group (36.2℃ ± 0.4℃ versus 35.9℃ ± 0.4℃, *P*<0.05). Furthermore, the OB group showed a significantly lower incidence of postoperative hypothermia than the UB group (*P*<0.05). Although the incidence of postoperative shivering was not significantly different between the two groups, 9 of 47 patients in the UB group suffered postoperative shivering, compared with only 4 of 49 in the OB group. These postoperative data are summarized in Table 3. Throughout the study period, the difference in the mean body temperature between the two groups never exceeded the clinically significant threshold (0.6°C) defined before the study.

**Table 3:** Comparison of Postoperative body temperature, Incidence of Postoperative hypothermia and shivering

	UB group (*n*=47)	OB group (*n*=49)	*P* value*
postoperative body temperature, (℃)	35.9 ± 0.4	36.2 ± 0.4	<0.001
Postoperative hypothermia,n (%)	27 (57.4%)	16 (32.7%)	0.023
Postoperative Shivering,n (%)	9 (19.1%)	4 (8.2%)	0.143

Data are expressed as means ± SDs.

^*^The *P* value for the *t* test and Fisher exact test is set at 0.05

## Discussion

In this randomized controlled trial, we investigated the effect of two different uses of underbody FAW blankets in preventing IPH in patients undergoing arthroscopic shoulder surgery. Our study showed that the use of a FAW blanket placed over the body was superior to the use of that placed under the body in preventing IPH. Although the body temperature was significantly different between the two groups at all time points from 45 minutes of using FAW onward, the difference never exceeded 0.6 ℃, the null hypothesis could not be rejected. The difference in body temperature was not clinically significant. Nevertheless, during the postoperative phase, 57.4% of the patients in the UB group suffered hypothermia but only 32.7% of the patients in the OB group suffered hypothermia.

Patients undergoing arthroscopic shoulder surgery are more prone to suffering IPH [22]. This is due to the use of large quantities of irrigation solution to ensure adequate intra-articular visualization. The shoulder is located close to the thorax and great vessels, and the room-temperature irrigation solution passing through the operative region can remove core body heat, which may lead to a considerable decrease in CBT. Although previous studies showed that IPH in arthroscopic shoulder surgery could be prevented with the use of warm irrigation solution [23, 24], other similar studies found no difference between the use of room-temperature irrigation solution and warm irrigation solution [7, 25]. Warm irrigation solution facilitates vasodilatation and bleeding during arthroscopy, which leads to surgical field of view impairment and prolonged duration of surgery. In consideration of these factors, room-temperature irrigation solution has always been used for arthroscopic shoulder surgery in our hospital.

After the induction of general anaesthesia, CBT decreases so rapidly in the first hour that it cannot be explained by heat loss due to the environment alone [3]. A patient's temperature should be measured at 30 min intervals from the start of anesthesia induction to the end of surgery according to the NICE Clinical Guidelines [26]. CBT measurements are considered to be more reliable than peripheral area temperature measurements because they are not influenced by ambient temperature or any other external factors [5]. ARON recommends using one consistent site and method to monitor CBT throughout the perioperative phase when clinically feasible [5]. However, it is difficult to monitor CBT continuously and accurately. The most accurate sites that are commonly used for the direct measurement of CBT in the clinical setting, such as esophagus, pulmonary artery, and nasopharynx are also the most invasive. Therefore, the direct measurement of CBT is usually performed after general anesthesia, which makes it impossible to be applied throughout the perioperative period. In addition to the direct measurement of CBT, a direct estimate of CBT, which is acquired from some designated sites, is also recommended [26]. The axillary region is the most commonly used site for acquiring a direct estimate of CBT, and its accuracy has also been confirmed [20, 21, 26, 27]. The iThermonitor is a commercial thermometer, which provides a non-invasive, wireless and wearable temperature monitoring solution. Axillary temperature recorded by the iThermonitor could represent CBT well both in children and adults having non-cardiac surgery [20, 21], so it is also called the estimated core temperature [21] or near-core temperature [27] . All these features of the iThermonitor allowed us to continuously and accurately monitor variations in CBT during the study.

IPH should be prevented or treated by appropriate methods throughout the perioperative period [5]. Among all warming methods, FAW has been considered to be the most effective [7, 13]. It protects patients from IPH not only through heat conduction, but also by preventing radiant and convective heat loss. Before the study, we tested the effectiveness of the underbody FAW unit used in this study. We included 20 patients and randomly divided them into a control group and a FAW group (10 per group). The patients in the control group did not receive any active body warming methods, and the FAW group used the FAW unit, with the FAW blanker placed under the patients. The results of our pilot trial showed that the body temperature of patients after using the FAW unit for 90 minutes was significantly higher: 35.9℃ ± 0.4℃ in the FAW group versus 35.4℃ ± 0.4℃ in the control group, which was consistent with previous literature reports [28]. The FAW unit we used in this study has exhibited great potential in the prevention of IPH.

Both underbody and over-body FAW blankets can be used for heat maintenance during arthroscopic shoulder surgery. The underbody blanket can be placed on the operating table in advance and is suitable for nearly all types of surgery and patient position. Moreover, it exhibited adequate effects in preventing IPH [28, 29]. These advantages allow anesthesiologists and operating room nurses to focus on the patient and warming as soon as the patient arrives in the operating room, thus making it increasingly popular in clinical practice. In this study, we placed full access underbody FAW blankets in 2 different positions (under and over the patient's body) during arthroscopic shoulder surgery, and investigated the effects of the 2 different uses. Our results showed that FAW blankets placed over patients have more advantages in preventing IPH than those placed under patients. This is a contradiction to the findings in the previous studies of Gulia et al. [29] and Alparslan et al. [30], as they found that an underbody FAW blanket can be as effective as both over-body and upper body blankets in preventing IPH in lower abdominal surgery. A possible reason is that the over-body and upper body blankets may not be able to cover the large body surface area involved the surgical site in lower abdominal surgery. However, in arthroscopic shoulder surgery, nearly two-thirds of the body surface area can be covered and heated with a full body blanket, thus placing the underbody blanket over patients may enhance its warming effects. In addition, when the blanket was placed under the body of the patients, the patient’s natural pressure points and the positioning devices used for stabilizing the lateral decubitus position may compress the underbody blanket, and prevent heat transfer.

It should be noted that this study has limitations. We only compared the warming effects of two different placements of full access underbody FAW blankets. Over-body and upper body FAW blankets were not used in our study, although the upper body blanket is preferred in clinics [31] and has been validated to be more effective than lower body blankets in patients in the lateral decubitus position [32]. However, the full access blanket can cover a much greater area than the upper body blanket in arthroscopic shoulder surgery [18], and its warming effects are better in theory. The difference in warming effects between placing over-body blankets and underbody blankets over patients in arthroscopic shoulder surgery will be investigated in a future study. Additionally, we did not warm the IV fluids, but we restricted fluid administration during the operation, which may have minimized bias. Finally, to standardize the warming protocol and reduce the impact of airflow caused by FAW on disinfection of the surgical area, we did not initiate FAW as soon as the patients arrived in the operating room. This may have weakened the warming effects of FAW.

## Conclusion

The body temperature was significantly better with the use of underbody FAW blankets placed over patients than with them placed under patients. Although the differences in body temperature never reached the level of predefined clinical significance (0.6℃), the incidence of postoperative hypothermia in the OB group was much lower than that in the UB group. Therefore, placing underbody FAW blankets over patients is recommended for the prevention of IPH in patients undergoing arthroscopic shoulder surgery.

## Supplementary Information

**Additional file 1.**

## Data Availability

All data generated or analysed during this study are included in this published article and supplementary information files.

## Abbreviations

FAW: Forced-air warming
IPH: Inadvertent perioperative hypothermia
CBT: Core body temperature
NICE: National Institute for Health and Care Excellence
AORN: Association of Perioperative Registered Nurses
ASA: American Society of Anesthesiologists
BMI: Body mass index

## References

[1] Collins S, Budds M, Raines C, Hooper V (2019). Risk Factors for Perioperative Hypothermia: A Literature Review. J Perianesth Nurs.

[2] Yi J, Lei Y-J, Xu S-Y, Si Y-Y, Li S-Y, Xia Z-Y (2017). Intraoperative hypothermia and its clinical outcomes in patients undergoing general anesthesia: National study in China. PLoS One..

[3] Sessler DI (2016). Perioperative thermoregulation and heat balance. Lancet.

[4] Burns SM, Wojnakowski M, Piotrowski K, Caraffa G (2009). Unintentional hypothermia: implications for perianesthesia nurses. J Perianesth Nurs..

[5] Link T (2020). Guidelines in Practice: Hypothermia Prevention. Aorn j.

[6] Siddiqiui T, Pal KMI, Shaukat F, Mubashir H, Akbar Ali A, Malik MJA (2020). Association Between Perioperative Hypothermia and Surgical Site Infection After Elective Abdominal Surgery: A Prospective Cohort Study. Cureus..

[7] Ralte P, Mateu-Torres F, Winton J, Bardsley J, Smith M, Kent M (2020). Prevention of Perioperative Hypothermia: A Prospective, Randomized, Controlled Trial of Bair Hugger Versus Inditherm in Patients Undergoing Elective Arthroscopic Shoulder Surgery. Arthroscopy.

[8] Wagner D, Hooper V, Bankieris K, Johnson A (2021). The Relationship of Postoperative Delirium and Unplanned Perioperative Hypothermia in Surgical Patients. J Perianesth Nurs.

[9] Moellhoff N, Broer PN, Heidekrueger PI, Ninkovic M, Ehrl D (2021). Impact of Intraoperative Hypothermia on Microsurgical Free Flap Reconstructions. J Reconstr Microsurg.

[10] Ingram A, Harper M (2018). The health economic benefits of perioperative patient warming for prevention of blood loss and transfusion requirements as a consequence of inadvertent perioperative hypothermia. J Perioper Pract.

[11] Chaikof EL, Dalman RL, Eskandari MK, Jackson BM, Lee WA, Mansour MA (2018). The Society for Vascular Surgery practice guidelines on the care of patients with an abdominal aortic aneurysm. J Vasc Surg.

[12] Croke L (2019). Guideline for prevention of hypothermia. AORN J.

[13] John M, Crook D, Dasari K, Eljelani F, El-Haboby A, Harper CM (2016). Comparison of resistive heating and forced-air warming to prevent inadvertent perioperative hypothermia. Br J Anaesth.

[14] Nieh HC, Su S-F (2016). Meta-analysis: effectiveness of forced-air warming for prevention of perioperative hypothermia in surgical patients. J Adv Nurs.

[15] Bräuer A, Franke R, von Hammerstein-Equord A (2021). Conductive heating mattress leads to ECG changes that mimic pacemaker spikes. J Clin Monit Comput.

[16] Del Vecchio JJ, Chemes LN, Ghioldi ME, Dealbera ED, Daniel MP (2020). Comparison of two forced-air warming devices during foot and ankle surgery: a randomised controlled trial. J Perioper Pract.

[17] Buraimoh MA, Nash A, Howard B, Yousaf I, Koh E, Banagan K (2019). Effect of forced-air warming blanket position in elective lumbar spine surgery: Intraoperative body temperature and postoperative complications. Surg Neurol Int.

[18] Bräuer A, Quintel M (2009). Forced-air warming: technology, physical background and practical aspects. Curr Opin Anaesthesiol.

[19] Schulz KF, Altman DG, Moher D, CONSORT Group (2010). CONSORT 2010 statement: updated guidelines for reporting parallel group randomised trials. PLoS Med..

[20] Ji Y-T, Han D, Han L, Xie S-Y, Pan S-D (2021). The Accuracy of a Wireless Axillary Thermometer for Core Temperature Monitoring in Pediatric Patients Having Noncardiac Surgery: An Observational Study. J Perianesth Nurs.

[21] Pei L-J, Huang Y-G, Mao G-M, Sessler DI (2018). Axillary Temperature, as Recorded by the iThermonitor WT701, Well Represents Core Temperature in Adults Having Noncardiac Surgery. Anesth Analg.

[22] Cho CK, Chang M, Sung TY, Jee YS (2021). Incidence of postoperative hypothermia and its risk factors in adults undergoing orthopedic surgery under brachial plexus block: A retrospective cohort study. Int J Med Sci.

[23] Lin YB, Zhou CB, Liu ZY, Wu KZ, Chen SB, Wang Wh (2020). Room Temperature Versus Warm Irrigation Fluid Used for Patients Undergoing Arthroscopic Shoulder Surgery A Systematic Review and Meta Analysis. J Perianesth Nurs..

[24] Pan X-Y, Ye L-Y, Liu Z-T, Wen H, Hu Y-Z, Xu X-X (2015). Effect of irrigation fluid temperature on core body temperature and inflammatory response during arthroscopic shoulder surgery. Arch Orthop Trauma Surg.

[25] Oh JH, Kim JY, Chung SW, Park JS, Kim DH, Kim SH (2014). Warmed irrigation fluid does not decrease perioperative hypothermia during arthroscopic shoulder surgery. Arthroscopy.

[26] National Institute for Health and Care Excellence. Hypothermia: prevention and management in adults having surgery. In: National Institute for Health and Care Excellence: Guidelines. London: NICE; 2016. https://www.nice.org.uk/guidance/cg65/resources/hypothermia-prevention-and-management-in-adults-havingsurgery-pdf-975569636293. Accessed 20 Nov 2021. 32134602

[27] Sessler DI (2021). Perioperative Temperature Monitoring. Anesthesiology.

[28] Sumida H, Sugino S, Kuratani N, Konno D, Hasegawa JI, Yamauchi M (2019). Effect of forced-air warming by an underbody blanket on end-of-surgery hypothermia: a propensity score-matched analysis of 5063 patients. BMC Anesthesiol.

[29] Gulia A, Gupta N, Kumar V, Bhoriwal S, Malhotra RK, Bharti SJ (2021). Comparison of two forced air warming systems for prevention of intraoperative hypothermia in carcinoma colon patients: a prospective randomized study. J Clin Monit Comput.

[30] Alparslan V, Kus A, Hosten T, Ertargin M, Ozdamar D, Toker K (2018). Comparison of forced-air warming systems in prevention of intraoperative hypothermia. J Clin Monit Comput.

[31] Lee Y, Kim K (2021). Optimal Application of Forced Air Warming to Prevent Peri-Operative Hypothermia during Abdominal Surgery: A Systematic Review and Meta-Analysis. Int J Environ Res Public Health.

[32] Min SH, Yoon S, Yoon SH, Bahk JH, Seo JH (2018). Randomised trial comparing forced-air warming to the upper or lower body to prevent hypothermia during thoracoscopic surgery in the lateral decubitus position. Br J Anaesth.

