# Supplementary material for: Comparison of two different uses of underbody forced-air warming blankets for the prevention of hypothermia in patients undergoing arthroscopic shoulder surgery: a prospective randomized study
Source: BMC Anesthesiol. 2022 Feb 28;22:55. doi: 10.1186/s12871-022-01597-6 (PMC8883687; doi:10.1186/s12871-022-01597-6)
Supplement: Supplementary file 1 — Additional file 1. [file 12871_2022_1597_MOESM1_ESM.docx]

**Data in Table 1.**

| Patient  number | Group | Sex  (1=male,  2=female) | Age  (years) | Height  (cm) | Weight  (kg) | BMI  (kg/m2) | Infusion volume  (mL) | Irrigation time  (min) | Irrigation volume  (L) |
| --- | --- | --- | --- | --- | --- | --- | --- | --- | --- |
| 4 | UB | 2 | 48 | 154 | 60 | 25 | 950 | 103 | 23.10 |
| 7 | UB | 1 | 62 | 179 | 76 | 24 | 1000 | 112 | 25.20 |
| 9 | UB | 2 | 53 | 149 | 52 | 23 | 600 | 66 | 10.50 |
| 11 | UB | 2 | 52 | 149 | 51 | 23 | 650 | 71 | 12.00 |
| 12 | UB | 1 | 44 | 165 | 70 | 26 | 600 | 63 | 8.70 |
| 15 | UB | 1 | 58 | 168 | 82 | 29 | 800 | 86 | 18.30 |
| 16 | UB | 1 | 57 | 179 | 82 | 26 | 1000 | 116 | 27.30 |
| 18 | UB | 2 | 45 | 165 | 70 | 26 | 750 | 79 | 17.40 |
| 19 | UB | 1 | 65 | 156 | 57 | 23 | 800 | 99 | 20.40 |
| 20 | UB | 2 | 69 | 165 | 76 | 28 | 900 | 99 | 21.60 |
| 22 | UB | 2 | 20 | 170 | 77 | 27 | 800 | 93 | 19.20 |
| 23 | UB | 1 | 34 | 172 | 75 | 25 | 700 | 78 | 18.90 |
| 25 | UB | 1 | 60 | 170 | 72 | 25 | 600 | 62 | 9.00 |
| 26 | UB | 1 | 47 | 172 | 77 | 26 | 950 | 102 | 21.90 |
| 27 | UB | 1 | 37 | 175 | 61 | 20 | 700 | 90 | 16.50 |
| 28 | UB | 2 | 42 | 164 | 62 | 23 | 600 | 65 | 10.20 |
| 29 | UB | 1 | 40 | 180 | 77 | 24 | 750 | 83 | 17.70 |
| 30 | UB | 1 | 49 | 170 | 63 | 22 | 1000 | 115 | 23.70 |
| 31 | UB | 1 | 55 | 168 | 62 | 22 | 650 | 73 | 15.90 |
| 32 | UB | 2 | 51 | 155 | 72 | 30 | 800 | 99 | 21.90 |
| 34 | UB | 1 | 66 | 171 | 62 | 21 | 1000 | 120 | 28.50 |
| 36 | UB | 2 | 65 | 148 | 60 | 27 | 650 | 91 | 23.40 |
| 39 | UB | 2 | 50 | 156 | 68 | 28 | 650 | 78 | 17.40 |
| 38 | UB | 1 | 61 | 166 | 58 | 21 | 950 | 113 | 26.10 |
| 44 | UB | 2 | 67 | 149 | 58 | 26 | 600 | 64 | 9.30 |
| 48 | UB | 2 | 48 | 151 | 68 | 30 | 900 | 116 | 27.90 |
| 49 | UB | 1 | 52 | 161 | 75 | 29 | 750 | 98 | 24.60 |
| 50 | UB | 1 | 30 | 170 | 75 | 26 | 900 | 104 | 21.30 |
| 51 | UB | 1 | 29 | 165 | 63 | 23 | 650 | 79 | 13.50 |
| 52 | UB | 2 | 61 | 155 | 48 | 20 | 550 | 74 | 17.70 |
| 53 | UB | 1 | 63 | 160 | 72 | 28 | 850 | 103 | 22.50 |
| 57 | UB | 2 | 62 | 154 | 47 | 20 | 600 | 94 | 17.10 |
| 60 | UB | 1 | 63 | 160 | 62 | 24 | 850 | 101 | 22.20 |
| 63 | UB | 2 | 64 | 156 | 60 | 25 | 600 | 87 | 16.20 |
| 66 | UB | 2 | 54 | 157 | 69 | 28 | 800 | 99 | 19.50 |
| 72 | UB | 1 | 20 | 177 | 87 | 28 | 900 | 92 | 21.60 |
| 74 | UB | 2 | 55 | 158 | 71 | 28 | 700 | 89 | 18.30 |
| 78 | UB | 1 | 45 | 176 | 86 | 28 | 950 | 112 | 29.40 |
| 80 | UB | 2 | 54 | 150 | 50 | 22 | 550 | 81 | 16.50 |
| 81 | UB | 2 | 56 | 150 | 50 | 22 | 500 | 63 | 9.00 |
| 82 | UB | 1 | 67 | 164 | 71 | 26 | 900 | 108 | 23.40 |
| 83 | UB | 1 | 28 | 172 | 70 | 24 | 750 | 100 | 21.60 |
| 87 | UB | 1 | 70 | 158 | 60 | 24 | 850 | 103 | 22.80 |
| 89 | UB | 2 | 35 | 155 | 49 | 20 | 600 | 76 | 12.60 |
| 92 | UB | 2 | 57 | 160 | 50 | 20 | 600 | 78 | 17.10 |
| 96 | UB | 2 | 62 | 158 | 57 | 23 | 650 | 79 | 16.20 |
| 100 | UB | 1 | 57 | 177 | 89 | 28 | 950 | 105 | 24.30 |
| 1 | OB | 2 | 33 | 168 | 72 | 26 | 900 | 111 | 26.10 |
| 2 | OB | 2 | 70 | 155 | 64 | 27 | 1000 | 116 | 27.60 |
| 3 | OB | 2 | 49 | 163 | 50 | 19 | 850 | 107 | 23.40 |
| 5 | OB | 2 | 46 | 160 | 60 | 23 | 550 | 65 | 9.60 |
| 6 | OB | 2 | 68 | 150 | 67 | 30 | 900 | 109 | 22.20 |
| 8 | OB | 1 | 44 | 174 | 78 | 26 | 700 | 94 | 20.10 |
| 10 | OB | 2 | 63 | 146 | 48 | 22 | 750 | 87 | 18.00 |
| 17 | OB | 1 | 61 | 155 | 52 | 22 | 700 | 74 | 12.90 |
| 13 | OB | 2 | 53 | 159 | 57 | 23 | 600 | 68 | 10.50 |
| 14 | OB | 1 | 21 | 177 | 75 | 24 | 950 | 113 | 26.10 |
| 21 | OB | 2 | 36 | 175 | 72 | 24 | 750 | 87 | 16.80 |
| 24 | OB | 2 | 52 | 157 | 48 | 19 | 800 | 109 | 23.40 |
| 35 | OB | 2 | 65 | 154 | 55 | 23 | 650 | 95 | 19.80 |
| 37 | OB | 1 | 52 | 165 | 53 | 19 | 600 | 87 | 17.10 |
| 41 | OB | 1 | 21 | 170 | 62 | 21 | 950 | 112 | 27.00 |
| 42 | OB | 2 | 53 | 156 | 67 | 28 | 600 | 91 | 17.40 |
| 43 | OB | 1 | 44 | 182 | 75 | 23 | 650 | 78 | 10.80 |
| 45 | OB | 1 | 61 | 164 | 67 | 25 | 700 | 82 | 13.80 |
| 46 | OB | 2 | 53 | 158 | 72 | 29 | 750 | 88 | 15.30 |
| 55 | OB | 1 | 36 | 175 | 85 | 28 | 950 | 108 | 23.40 |
| 54 | OB | 1 | 63 | 167 | 65 | 23 | 600 | 104 | 22.80 |
| 56 | OB | 2 | 54 | 158 | 58 | 23 | 600 | 82 | 14.70 |
| 58 | OB | 2 | 51 | 160 | 66 | 26 | 700 | 85 | 17.10 |
| 59 | OB | 1 | 55 | 161 | 73 | 28 | 950 | 117 | 27.30 |
| 61 | OB | 2 | 59 | 158 | 65 | 26 | 900 | 96 | 19.50 |
| 62 | OB | 2 | 69 | 155 | 50 | 21 | 600 | 76 | 12.60 |
| 64 | OB | 1 | 56 | 168 | 65 | 23 | 850 | 91 | 20.70 |
| 65 | OB | 1 | 53 | 167 | 70 | 25 | 1000 | 119 | 30.00 |
| 67 | OB | 1 | 57 | 154 | 50 | 21 | 650 | 87 | 18.90 |
| 68 | OB | 2 | 67 | 157 | 48 | 19 | 700 | 80 | 15.60 |
| 69 | OB | 1 | 51 | 170 | 59 | 20 | 600 | 83 | 16.80 |
| 70 | OB | 2 | 65 | 155 | 55 | 23 | 700 | 78 | 12.90 |
| 71 | OB | 2 | 57 | 160 | 75 | 29 | 750 | 89 | 17.10 |
| 73 | OB | 1 | 39 | 174 | 76 | 25 | 700 | 98 | 18.00 |
| 76 | OB | 2 | 66 | 155 | 55 | 23 | 750 | 84 | 17.40 |
| 77 | OB | 2 | 63 | 155 | 51 | 21 | 750 | 99 | 20.10 |
| 79 | OB | 1 | 53 | 168 | 65 | 23 | 750 | 86 | 18.30 |
| 84 | OB | 2 | 55 | 158 | 55 | 22 | 650 | 78 | 15.00 |
| 85 | OB | 2 | 55 | 159 | 60 | 24 | 550 | 62 | 8.10 |
| 86 | OB | 2 | 66 | 154 | 70 | 30 | 950 | 108 | 22.80 |
| 88 | OB | 2 | 49 | 155 | 72 | 30 | 750 | 72 | 12.90 |
| 90 | OB | 1 | 35 | 174 | 77 | 25 | 500 | 72 | 11.70 |
| 91 | OB | 1 | 33 | 165 | 68 | 25 | 950 | 106 | 22.80 |
| 93 | OB | 2 | 37 | 166 | 70 | 25 | 650 | 81 | 17.40 |
| 94 | OB | 1 | 28 | 168 | 60 | 21 | 850 | 87 | 18.60 |
| 95 | OB | 1 | 56 | 168 | 67 | 24 | 700 | 99 | 21.30 |
| 97 | OB | 2 | 57 | 154 | 68 | 29 | 550 | 76 | 12.90 |
| 98 | OB | 1 | 68 | 177 | 75 | 24 | 900 | 83 | 16.80 |
| 99 | OB | 1 | 54 | 160 | 55 | 21 | 1000 | 107 | 22.20 |

**Data in Table 2 and Figure 3.**

| Patient  number | Group | Ta (℃) | Tb (℃) | T0 (℃) | T15 (℃) | T30 (℃) | T45 (℃) | T60 (℃) | T75 (℃) | T90 (℃) |
| --- | --- | --- | --- | --- | --- | --- | --- | --- | --- | --- |
| 4 | UB | 36.50 | 36.40 | 36.30 | 36.00 | 35.80 | 35.70 | 35.50 | 35.40 | 35.30 |
| 7 | UB | 36.30 | 36.20 | 36.00 | 35.80 | 35.60 | 35.50 | 35.40 | 35.30 | 35.20 |
| 9 | UB | 36.50 | 36.40 | 36.20 | 36.00 | 35.90 | 35.80 | 35.70 | 35.80 | 36.00 |
| 11 | UB | 36.80 | 36.60 | 36.40 | 36.30 | 36.20 | 36.10 | 35.90 | 35.90 | 36.10 |
| 12 | UB | 36.50 | 36.40 | 36.20 | 36.00 | 35.80 | 35.60 | 35.50 | 35.60 | 35.50 |
| 15 | UB | 36.50 | 36.50 | 36.40 | 36.10 | 36.00 | 35.80 | 35.70 | 35.50 | 35.60 |
| 16 | UB | 36.90 | 36.80 | 36.70 | 36.40 | 36.30 | 36.10 | 36.00 | 35.80 | 35.60 |
| 18 | UB | 37.20 | 37.10 | 36.80 | 36.60 | 36.40 | 36.20 | 36.10 | 36.00 | 36.00 |
| 19 | UB | 36.40 | 36.40 | 36.10 | 36.00 | 35.80 | 35.70 | 35.60 | 35.40 | 35.50 |
| 20 | UB | 36.30 | 36.20 | 35.80 | 35.50 | 35.30 | 35.20 | 35.10 | 35.00 | 34.90 |
| 22 | UB | 37.00 | 36.90 | 36.70 | 36.60 | 36.40 | 36.30 | 36.20 | 36.10 | 36.30 |
| 23 | UB | 36.80 | 36.70 | 36.50 | 36.30 | 36.20 | 36.10 | 35.90 | 35.80 | 36.00 |
| 25 | UB | 36.60 | 36.50 | 36.30 | 36.00 | 35.90 | 35.70 | 35.80 | 35.90 | 36.20 |
| 26 | UB | 36.50 | 36.50 | 36.30 | 36.10 | 35.80 | 35.60 | 35.40 | 35.40 | 35.30 |
| 27 | UB | 36.90 | 36.90 | 36.70 | 36.50 | 36.40 | 36.30 | 36.10 | 36.10 | 35.90 |
| 28 | UB | 36.70 | 36.60 | 36.30 | 36.10 | 35.90 | 35.70 | 35.60 | 35.80 | 36.00 |
| 29 | UB | 37.30 | 37.20 | 36.90 | 36.80 | 36.50 | 36.40 | 36.20 | 36.10 | 36.00 |
| 30 | UB | 36.90 | 36.80 | 36.70 | 36.50 | 36.30 | 36.20 | 36.20 | 36.10 | 35.90 |
| 31 | UB | 36.50 | 36.40 | 36.20 | 35.90 | 35.70 | 35.60 | 35.50 | 35.40 | 35.60 |
| 32 | UB | 37.10 | 37.00 | 36.80 | 36.60 | 36.40 | 36.20 | 36.10 | 36.00 | 35.90 |
| 34 | UB | 37.20 | 37.10 | 37.00 | 36.70 | 36.50 | 36.40 | 36.20 | 36.00 | 36.10 |
| 36 | UB | 36.40 | 36.30 | 36.00 | 35.80 | 35.60 | 35.50 | 35.40 | 35.50 | 35.30 |
| 39 | UB | 36.20 | 36.20 | 35.90 | 35.60 | 35.40 | 35.30 | 35.20 | 35.30 | 35.40 |
| 38 | UB | 36.50 | 36.50 | 36.30 | 36.00 | 35.80 | 35.70 | 35.60 | 35.60 | 35.50 |
| 44 | UB | 37.10 | 37.00 | 36.90 | 36.60 | 36.40 | 36.30 | 36.10 | 36.00 | 36.10 |
| 48 | UB | 37.20 | 37.20 | 36.90 | 36.70 | 36.50 | 36.30 | 36.40 | 36.50 | 36.30 |
| 49 | UB | 37.60 | 36.90 | 36.70 | 36.50 | 36.40 | 36.30 | 36.10 | 36.00 | 35.80 |
| 50 | UB | 37.20 | 37.10 | 36.90 | 36.70 | 36.50 | 36.40 | 36.30 | 36.20 | 36.10 |
| 51 | UB | 37.80 | 37.70 | 37.50 | 37.40 | 37.20 | 37.00 | 36.90 | 36.80 | 36.90 |
| 52 | UB | 36.40 | 36.30 | 36.10 | 35.80 | 35.60 | 35.40 | 35.30 | 35.40 | 35.50 |
| 53 | UB | 36.80 | 36.70 | 36.40 | 36.20 | 36.10 | 35.90 | 35.80 | 35.80 | 35.60 |
| 57 | UB | 36.40 | 36.30 | 36.10 | 35.90 | 35.70 | 35.60 | 35.50 | 35.50 | 35.40 |
| 60 | UB | 36.40 | 35.30 | 36.10 | 35.90 | 35.70 | 35.60 | 35.60 | 35.50 | 35.40 |
| 63 | UB | 36.30 | 36.20 | 36.00 | 35.70 | 35.60 | 35.40 | 35.50 | 35.40 | 35.50 |
| 66 | UB | 36.60 | 36.50 | 36.20 | 36.10 | 35.90 | 35.70 | 35.60 | 35.50 | 35.70 |
| 72 | UB | 37.40 | 37.30 | 37.10 | 36.90 | 36.70 | 36.50 | 36.50 | 36.60 | 36.40 |
| 74 | UB | 36.20 | 36.10 | 35.90 | 35.70 | 35.60 | 35.60 | 35.50 | 35.40 | 35.40 |
| 78 | UB | 36.90 | 36.80 | 36.60 | 36.40 | 36.30 | 36.20 | 36.10 | 36.00 | 36.00 |
| 80 | UB | 36.60 | 36.50 | 36.30 | 36.10 | 35.90 | 35.80 | 35.70 | 35.60 | 35.60 |
| 81 | UB | 36.60 | 36.50 | 36.30 | 36.10 | 35.90 | 35.70 | 35.60 | 35.80 | 35.90 |
| 82 | UB | 36.30 | 36.20 | 36.10 | 35.90 | 35.70 | 35.50 | 35.40 | 35.20 | 35.30 |
| 83 | UB | 36.80 | 36.80 | 36.60 | 36.30 | 36.20 | 36.10 | 36.00 | 35.90 | 36.10 |
| 87 | UB | 36.50 | 36.40 | 36.00 | 35.70 | 35.50 | 35.50 | 35.40 | 35.30 | 35.50 |
| 89 | UB | 37.10 | 37.10 | 36.90 | 36.70 | 36.50 | 36.40 | 36.30 | 36.20 | 36.30 |
| 92 | UB | 36.80 | 36.70 | 36.60 | 36.40 | 36.10 | 36.10 | 35.90 | 35.80 | 35.70 |
| 96 | UB | 36.60 | 36.50 | 36.20 | 36.10 | 35.70 | 35.60 | 35.40 | 35.30 | 35.40 |
| 100 | UB | 36.40 | 36.30 | 36.10 | 36.00 | 35.90 | 35.80 | 35.80 | 35.70 | 35.80 |
| 1 | OB | 36.90 | 36.80 | 36.60 | 36.40 | 36.20 | 36.10 | 35.90 | 35.90 | 35.70 |
| 2 | OB | 36.80 | 36.70 | 36.40 | 36.20 | 36.00 | 35.90 | 36.00 | 35.90 | 36.00 |
| 3 | OB | 37.20 | 37.10 | 36.90 | 36.90 | 36.70 | 36.60 | 36.50 | 36.40 | 36.30 |
| 5 | OB | 36.50 | 36.40 | 35.20 | 36.10 | 35.80 | 35.90 | 36.00 | 35.90 | 36.10 |
| 6 | OB | 36.10 | 36.00 | 35.70 | 35.50 | 35.40 | 35.40 | 35.30 | 35.40 | 35.50 |
| 8 | OB | 37.20 | 37.10 | 36.90 | 36.70 | 36.60 | 36.40 | 36.50 | 36.60 | 36.60 |
| 10 | OB | 36.60 | 36.50 | 36.20 | 36.00 | 35.90 | 35.90 | 35.70 | 35.70 | 35.90 |
| 17 | OB | 36.40 | 36.30 | 36.00 | 35.90 | 35.70 | 35.60 | 35.60 | 35.70 | 35.90 |
| 13 | OB | 36.70 | 36.60 | 36.40 | 36.10 | 36.00 | 36.00 | 35.90 | 36.00 | 36.10 |
| 14 | OB | 37.30 | 37.20 | 37.00 | 36.90 | 36.70 | 36.60 | 36.60 | 36.60 | 36.80 |
| 21 | OB | 36.80 | 36.70 | 36.50 | 36.30 | 36.20 | 36.10 | 36.10 | 36.10 | 36.20 |
| 24 | OB | 36.50 | 36.50 | 36.20 | 36.00 | 35.80 | 35.90 | 36.00 | 36.10 | 36.10 |
| 35 | OB | 36.40 | 36.30 | 36.10 | 35.90 | 35.70 | 35.60 | 35.40 | 35.50 | 35.50 |
| 37 | OB | 36.80 | 36.70 | 36.40 | 36.20 | 36.10 | 36.00 | 35.90 | 35.80 | 35.70 |
| 41 | OB | 37.20 | 37.20 | 37.00 | 36.80 | 36.80 | 36.70 | 36.50 | 36.50 | 36.60 |
| 42 | OB | 36.60 | 36.60 | 36.40 | 36.20 | 36.00 | 36.00 | 35.90 | 35.80 | 35.90 |
| 43 | OB | 36.70 | 36.70 | 36.50 | 36.30 | 36.20 | 36.20 | 36.10 | 36.00 | 36.10 |
| 45 | OB | 36.30 | 36.20 | 36.00 | 35.90 | 35.70 | 35.80 | 35.60 | 35.70 | 35.80 |
| 46 | OB | 36.80 | 36.70 | 36.40 | 36.30 | 36.30 | 36.20 | 36.10 | 36.20 | 36.30 |
| 55 | OB | 37.70 | 37.70 | 37.30 | 37.10 | 37.10 | 37.00 | 37.00 | 36.90 | 36.80 |
| 54 | OB | 36.50 | 36.30 | 36.10 | 36.10 | 36.10 | 36.00 | 35.90 | 36.00 | 36.10 |
| 56 | OB | 36.50 | 36.30 | 36.10 | 35.80 | 35.80 | 35.90 | 35.80 | 35.90 | 36.10 |
| 58 | OB | 37.20 | 37.20 | 37.10 | 36.80 | 36.80 | 36.70 | 36.70 | 36.60 | 36.60 |
| 59 | OB | 36.60 | 36.50 | 36.30 | 36.00 | 35.80 | 35.60 | 35.40 | 35.50 | 35.60 |
| 61 | OB | 36.70 | 36.70 | 36.50 | 36.30 | 36.20 | 36.20 | 36.10 | 36.10 | 36.00 |
| 62 | OB | 36.20 | 36.10 | 36.00 | 35.80 | 35.60 | 35.50 | 35.40 | 35.30 | 35.30 |
| 64 | OB | 36.50 | 36.50 | 36.30 | 36.10 | 36.00 | 35.90 | 36.00 | 36.10 | 36.20 |
| 65 | OB | 36.90 | 36.80 | 36.60 | 36.30 | 36.20 | 36.10 | 36.00 | 35.90 | 35.80 |
| 67 | OB | 36.70 | 36.60 | 36.50 | 36.30 | 36.10 | 36.00 | 35.90 | 35.80 | 35.70 |
| 68 | OB | 36.80 | 36.70 | 36.50 | 36.20 | 36.10 | 36.00 | 35.80 | 36.00 | 36.10 |
| 69 | OB | 37.00 | 36.90 | 36.80 | 36.60 | 36.40 | 36.40 | 36.50 | 36.40 | 36.50 |
| 70 | OB | 36.50 | 36.40 | 36.40 | 36.20 | 36.10 | 36.10 | 36.00 | 36.20 | 36.20 |
| 71 | OB | 36.50 | 36.40 | 36.20 | 36.10 | 36.10 | 36.00 | 36.00 | 35.90 | 36.00 |
| 73 | OB | 37.30 | 37.20 | 37.00 | 36.90 | 36.90 | 36.90 | 36.80 | 36.70 | 36.80 |
| 76 | OB | 36.70 | 36.70 | 36.50 | 36.40 | 36.20 | 36.10 | 36.00 | 35.90 | 35.80 |
| 77 | OB | 36.40 | 36.30 | 36.00 | 35.90 | 35.80 | 35.80 | 36.00 | 36.20 | 36.30 |
| 79 | OB | 36.60 | 36.60 | 36.30 | 36.10 | 35.90 | 35.90 | 35.80 | 35.90 | 36.00 |
| 84 | OB | 37.00 | 36.90 | 36.70 | 36.50 | 36.40 | 36.30 | 36.10 | 36.00 | 36.20 |
| 85 | OB | 36.50 | 36.30 | 36.00 | 35.90 | 35.80 | 35.70 | 35.70 | 35.80 | 36.00 |
| 86 | OB | 36.50 | 36.40 | 36.20 | 36.00 | 35.90 | 35.80 | 35.60 | 35.60 | 35.50 |
| 88 | OB | 36.70 | 36.60 | 36.50 | 36.50 | 36.30 | 36.20 | 36.10 | 36.20 | 36.30 |
| 90 | OB | 37.30 | 37.20 | 37.10 | 37.00 | 36.80 | 36.70 | 36.50 | 36.60 | 36.80 |
| 91 | OB | 37.10 | 37.10 | 36.90 | 36.80 | 36.60 | 36.50 | 36.60 | 36.50 | 36.50 |
| 93 | OB | 36.90 | 36.80 | 36.60 | 36.60 | 36.20 | 36.20 | 36.10 | 36.30 | 36.40 |
| 94 | OB | 36.90 | 36.90 | 36.70 | 36.70 | 36.40 | 36.30 | 36.20 | 36.30 | 36.20 |
| 95 | OB | 36.30 | 36.20 | 36.10 | 35.90 | 35.80 | 35.80 | 35.80 | 35.70 | 35.80 |
| 97 | OB | 36.70 | 36.60 | 36.30 | 36.10 | 36.10 | 36.00 | 36.00 | 36.00 | 36.10 |
| 98 | OB | 36.70 | 36.60 | 36.40 | 36.50 | 36.30 | 36.20 | 36.10 | 36.00 | 35.90 |
| 99 | OB | 36.80 | 36.70 | 36.50 | 36.40 | 36.30 | 36.20 | 36.30 | 36.50 | 36.60 |

**Data in Table 3.**

| Patient  number | Group | Postoperative CBT (℃) | Shivering |
| --- | --- | --- | --- |
| 4 | UB | 35.40 | Yes |
| 7 | UB | 35.20 | Yes |
| 9 | UB | 36.40 |  |
| 11 | UB | 36.30 |  |
| 12 | UB | 35.90 |  |
| 15 | UB | 35.80 |  |
| 16 | UB | 35.50 |  |
| 18 | UB | 36.20 |  |
| 19 | UB | 35.70 |  |
| 20 | UB | 35.20 | Yes |
| 22 | UB | 36.40 |  |
| 23 | UB | 36.30 |  |
| 25 | UB | 36.40 |  |
| 26 | UB | 35.50 | Yes |
| 27 | UB | 36.00 |  |
| 28 | UB | 36.30 |  |
| 29 | UB | 36.20 |  |
| 30 | UB | 35.70 |  |
| 31 | UB | 35.90 |  |
| 32 | UB | 36.10 |  |
| 34 | UB | 36.30 |  |
| 36 | UB | 35.20 | Yes |
| 39 | UB | 35.60 |  |
| 38 | UB | 35.30 | Yes |
| 44 | UB | 36.40 |  |
| 48 | UB | 36.20 |  |
| 49 | UB | 36.00 |  |
| 50 | UB | 35.80 |  |
| 51 | UB | 36.80 |  |
| 52 | UB | 35.80 |  |
| 53 | UB | 35.80 |  |
| 57 | UB | 35.60 |  |
| 60 | UB | 35.20 | Yes |
| 63 | UB | 35.80 |  |
| 66 | UB | 35.90 |  |
| 72 | UB | 36.60 |  |
| 74 | UB | 35.50 |  |
| 78 | UB | 36.10 |  |
| 80 | UB | 35.70 |  |
| 81 | UB | 36.20 |  |
| 82 | UB | 35.30 | Yes |
| 83 | UB | 36.20 |  |
| 87 | UB | 35.40 |  |
| 89 | UB | 36.50 |  |
| 92 | UB | 36.00 | Yes |
| 96 | UB | 35.60 |  |
| 100 | UB | 35.90 |  |
| 1 | OB | 35.60 |  |
| 2 | OB | 35.90 |  |
| 3 | OB | 36.60 |  |
| 5 | OB | 36.40 |  |
| 6 | OB | 35.70 |  |
| 8 | OB | 36.60 |  |
| 10 | OB | 36.10 |  |
| 17 | OB | 36.10 |  |
| 13 | OB | 36.40 |  |
| 14 | OB | 36.90 |  |
| 21 | OB | 36.50 |  |
| 24 | OB | 36.30 |  |
| 35 | OB | 35.70 |  |
| 37 | OB | 35.70 |  |
| 41 | OB | 36.60 |  |
| 42 | OB | 35.90 |  |
| 43 | OB | 36.30 |  |
| 45 | OB | 36.00 |  |
| 46 | OB | 36.50 |  |
| 55 | OB | 36.70 |  |
| 54 | OB | 36.20 |  |
| 56 | OB | 36.30 |  |
| 58 | OB | 36.70 |  |
| 59 | OB | 35.50 | Yes |
| 61 | OB | 35.90 |  |
| 62 | OB | 35.40 | Yes |
| 64 | OB | 36.40 |  |
| 65 | OB | 35.70 |  |
| 67 | OB | 35.80 |  |
| 68 | OB | 36.20 |  |
| 69 | OB | 36.60 |  |
| 70 | OB | 36.40 |  |
| 71 | OB | 36.20 |  |
| 73 | OB | 36.80 |  |
| 76 | OB | 35.90 |  |
| 77 | OB | 36.30 |  |
| 79 | OB | 36.10 |  |
| 84 | OB | 36.50 |  |
| 85 | OB | 36.40 |  |
| 86 | OB | 35.40 | Yes |
| 88 | OB | 36.30 |  |
| 90 | OB | 36.80 |  |
| 91 | OB | 36.70 |  |
| 93 | OB | 36.60 | Yes |
| 94 | OB | 36.50 |  |
| 95 | OB | 35.80 |  |
| 97 | OB | 36.30 |  |
| 98 | OB | 35.80 |  |
| 99 | OB | 36.80 |  |
